# Microplastics Pollution as an Invisible Potential Threat to Food Safety and Security, Policy Challenges and the Way Forward

**DOI:** 10.3390/ijerph17249591

**Published:** 2020-12-21

**Authors:** Sunusi Usman, Ahmad Faizal Abdull Razis, Khozirah Shaari, Mohammad Noor Azmai Amal, Mohd Zamri Saad, Nurulfiza Mat Isa, Muhammad Farhan Nazarudin, Syaizwan Zahmir Zulkifli, Jumria Sutra, Musa Adamu Ibrahim

**Affiliations:** 1 Natural Medicines and Products Research Laboratory, Institute of Bioscience, Universiti Putra Malaysia, 43400 UPM Serdang, Selangor, Malaysia; usunusi.bch@buk.edu.ng (S.U.); khozirah@upm.edu.my (K.S.); 2 Department of Food Science, Faculty of Food Science and Technology, Universiti Putra Malaysia, 43400 UPM Serdang, Selangor, Malaysia; 3 Department of Chemistry, Faculty of Science, Universiti Putra Malaysia, 43400 UPM Serdang, Selangor, Malaysia; 4 Department of Biology, Faculty of Science, Universiti Putra Malaysia, 43400 UPM Serdang, Selangor, Malaysia; mnamal@upm.edu.my (M.N.A.A.); syaizwan@upm.edu.my (S.Z.Z.); jumriasutra@gmail.com (J.S.); maibrahim@unimaid.edu.ng (M.A.I.); 5 Aquatic Animal Health and Therapeutics Laboratory (Aqua Health), Institute of Bioscience, Universiti Putra Malaysia, 43400 UPM Serdang, Selangor, Malaysia; mzamri@upm.edu.my (M.Z.S.); m_farhannaza@upm.edu.my (M.F.N.); 6 Department of Veterinary Laboratory Diagnosis, Faculty of Veterinary Medicine, Universiti Putra Malaysia, 43400 UPM Serdang, Selangor, Malaysia; 7 Department of Cell and Molecular Biology, Faculty of Biotechnology and Bimolecular Sciences, Universiti Putra Malaysia, 43400 UPM Serdang, Selangor, Malaysia; nurulfiza@upm.edu.my; 8 Laboratory of Vaccines and Biomolecules (VacBio), Institute of Bioscience, Universiti Putra Malaysia, 43400 UPM Serdang, Selangor, Malaysia

**Keywords:** microplastics, food safety, food security, exposure, toxicity, policies

## Abstract

Technological advances, coupled with increasing demands by consumers, have led to a drastic increase in plastic production. After serving their purposes, these plastics reach our water bodies as their destination and become ingested by aquatic organisms. This ubiquitous phenomenon has exposed humans to microplastics mostly through the consumption of sea food. This has led the World Health Organization (WHO) to make an urgent call for the assessment of environmental pollution due to microplastics and its effect on human health. This review summarizes studies between 1999 and 2020 in relation to microplastics in aquatic ecosystems and human food products, their potential toxic effects as elicited in animal studies, and policies on their use and disposal. There is a paucity of information on the toxicity mechanisms of microplastics in animal studies, and despite their documented presence in food products, no policy has been in place so far, to monitor and regulates microplastics in commercial foods meant for human consumption. Although there are policies and regulations with respect to plastics, these are only in a few countries and in most instances are not fully implemented due to socioeconomic reasons, so they do not address the problem across the entire life cycle of plastics from production to disposal. More animal research to elucidate pathways and early biomarkers of microplastic toxicity that can easily be detected in humans is needed. This is to create awareness and influence policies that will address this neglected threat to food safety and security.

## 1. Introduction

Manmade plastic waste entering the oceans are mostly made from synthetic and semi synthetic polymers. The oceans are invaluable as they provide energy, food and water. Alteration in the marine ecosystem by these plastic litters can have a global harmful effect [1]. As of 2010, 275 million metric tons (MT) of plastic was generated by 192 coastal countries, and of this 4.8 to 12.7 million MT entered the ocean [2]. A country’s contribution to plastic marine debris is determined by its population size and the standard and efficiency of its waste management systems. It has been postulated that if the standard of plastic waste management systems is not improved by the year 2025, the quantity of plastic waste that will be available to get into the oceans will increase by an order of magnitude [2]. Microplastics have been found in all seas [3] and marine environments [4]. Microplastics have become pollutants of environmental concern because little is known about their effects on human health, despite reports of their presence in food and air [5].

Knowledge regarding the negative health effects of consuming microplastic-containing marine organisms is lacking, difficult to ascertain and often controversial [6]. Activism with little or no resistance from political and corporate organizations together with sound and adequate scientific evidence of the negative effect of plastic pollution is dearly needed to strengthen and disseminate new environmental policies and norms to curtail the ravaging effect of marine plastic pollution [7].

**Figure 1:**
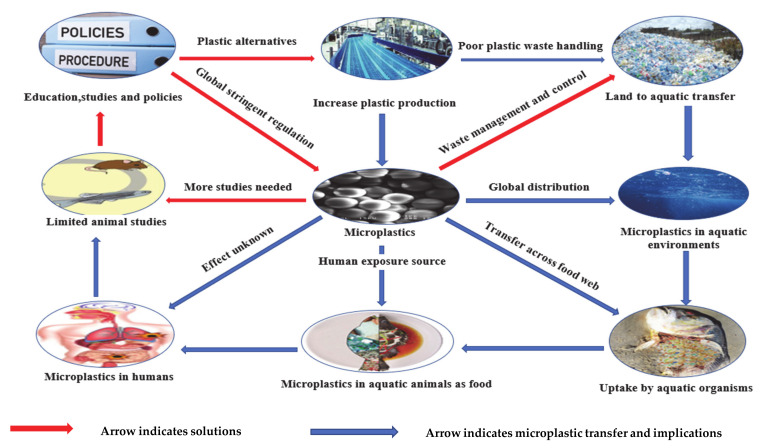
Pathways, health concerns and possible solutions to microplastic pollution.

As shown in Figure 1, the drastic increase in plastic production primarily as microplastics or larger plastics that degrade to generate secondary microplastics, coupled with their poor handling as waste, enables them to enter every waterbody. In aquatic environments, marine organisms ingest microplastics and they become transferred across the food chain. Humans become exposed to microplastics mainly via the consumption of sea food with unknown effects, prompting limited animal studies on the effects of microplastics. Thus, this requires further studies that will give more information to help to ascertain human health risk, educate consumers, provide plastic alternatives and influence policies that will control plastic production and waste management.

## 2. Microplastic Sources

Advances in science and technology related to new synthetic chemicals have drastically boosted the production of plastics, and this has made plastic an important commodity of our modern time [8]. Microplastics, which are plastics of a size smaller than 5 mm, have become objects of concern in the ocean and the aquatic environment generally. They are mostly synthesized from polyethylene (PE), polypropylene (PP), polystyrene (PS), polyethylene terephthalate (PET) and polyesters, and they reach the aquatic environment primarily as microbeads (<1 mm) from cosmetics, cleaning agents, broken fragments that result from the process of washing clothes, or secondarily as degraded plastic litter and debris [9].

Microplastics are often regarded as plastics whose longest diameter is less than 5 mm by most authors, although it has been proposed to only include actual plastics in the micrometer size and to adjust the size to less than 1 mm [10,11], yet the upper limit of 5 mm is still considered because plastics of this size can be ingested by marine organisms [12]. Primary microplastics in the marine environment are produced as micro sized, such as cosmetic rubbers and abrasive beads used in sand blasting, or are secondarily generated as a result of the disintegration of larger plastics, both of which get into the aquatic environment mostly as waste discharge or accidental spillages [13]. The secondary microplastics are generated through the effects of various processes, which can be physical (temperature, weather, mechanical forces), photodegradation (UV-light), biological (bacteria, fungi, algae) and lastly through chemical degradation via oxidation. The other processes of degradation alter the physiochemical properties of the polymer, making it brittle [14], and then mechanical degradation, which is the most important as far as plastic in the aquatic environment is concerned, breaks them into smaller sizes between 1 µm to 5000 µm—a size regarded as a microplastic [15]. These microplastics are said to undergo further degradation in some instances to give nano plastics [16]. The source and relative abundance of microplastics vary between regions depending on their waste management capabilities and efficiency; affluent regions tend to have much more of a problem with primary microplastics, such as those from cosmetics, and these plastics are mostly from land based sources which have access to marine environments via rivers and other routes [17,18]. Individuals utilizing facial scrubs have limited knowledge of them containing plastic particles and the beads in these products have a relatively small size, enabling them to bypass filtration processes in certain waste water treatment facilities [19].

**Figure 2:**
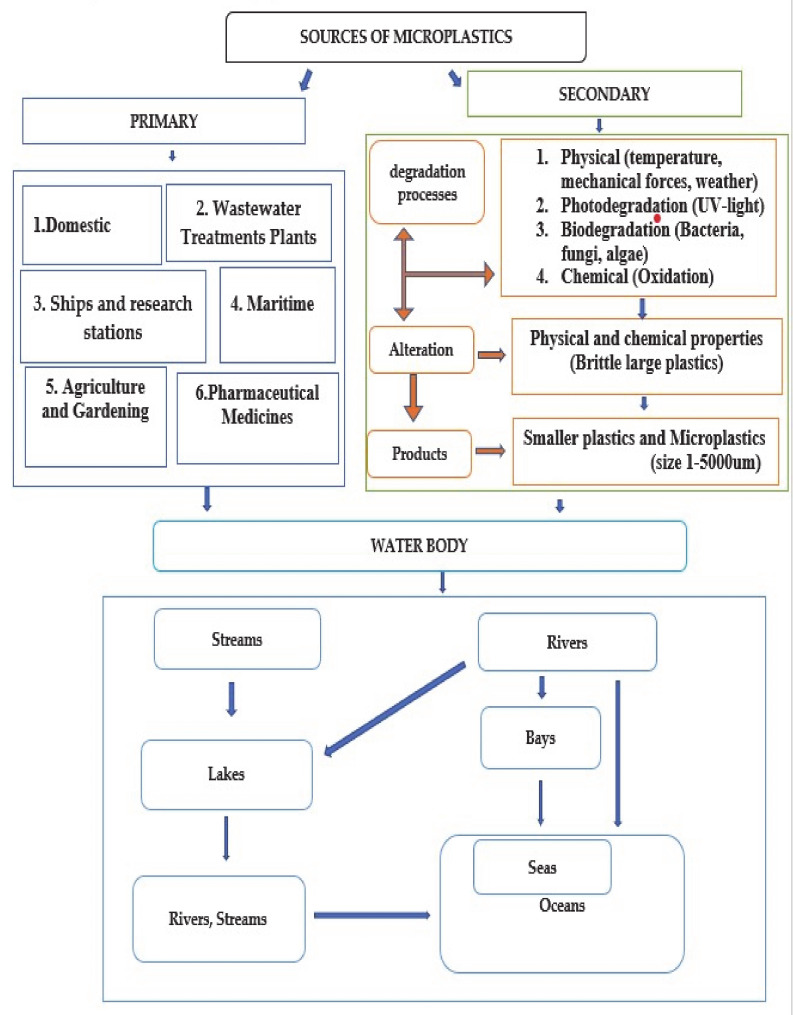
Sources and destinations of microplastics.

Ships and scientific research stations significantly contribute to microplastic pollution, especially at a local scale [20]. Mariculture activities such as enclosure, raft and cage culture, which use various types of microplastics, have been a contributing factor to microplastics in the marine environment [21]. Sewage sludge discharge contributes significantly to plastic pollution in the environment, as seen in eastern and western China where an average of 22.7 ± 12.1 × 10^3^ particles per kilogram of dry sludge was detected, with most of them being polyolefin, acrylic fibers, polyethylene and polyamide. An average of 1.56 × 10^14^ particles per year of microplastics enters the natural environment based on total sludge production in China [22]. Enormous and widely distributed quantities of microplastics in fresh water are said to be contributed by waste water treatment plants; this is evident by the increasing concentration of microplastics downstream, and this is in addition to the contribution of microplastics from other sources aside from waste water treatment plants [23]. Organic fertilizers used in agriculture and gardening worldwide tend to be a neglected source of microplastics, and this includes fertilizers pretreated by both composting and fermentation [24]. As shown in Figure 2, microplastics from domestic sources (cosmetics, cleansing agents, laundry, facial scrubs), wastewater treatment plants, ships, research stations, maritime (fishing and shipping), agricultural activities and pharmaceutical products such as medicines have ended up in our water bodies. Additionally, larger plastics have been altered and degraded into microplastics over time and have become deposited in our rivers, seas, and oceans.

## 3. Microplastic Distribution in Aquatic Ecosystems

Microplastics are distributed in both the sea and freshwater [25]. They are found to have reached most marine environments. Different habitats of deep sea sediments ranging in depth from 1100 m to 5000 m were found to contain microplastics [26], and they are also found in the remotest locations of deep sea water [27]. The abundance of microplastics in freshwater is similar to that of sea water [28], with varied distribution [29]. Municipal waste water effluents from 17 different treatment facilities in the United States were found to release over 4 million particles per facility per day, with fibers and fragments as the most common type of particle [30]. Rice-fish co-culture farming system was found to have contributed to microplastic pollution, as evident from the presence of microplastics in samples of water, soil, and animals in both rice planting and non-planting periods, with the most predominant microplastics being white and translucent poly-ethylene and polypropylene fibers of smaller sizes of less than 1 mm [31]. These microplastics that come from various sources enter the water ways, mostly ending up in freshwater before finally reaching the seas and oceans; however, this depends on the proximity of the source to either freshwater or seawater. This has led to the global distribution of microplastics in the aquatic ecosystem. As shown in Table 1, microplastics are found in seawater, freshwater and sediment across the globe. The predominant types found were polyethylene, polypropylene, and polystyrene, most in the size range of less than 5 mm. Thus, this shows the ubiquitous nature of this pollutant in our water bodies at a size that can easily be ingested and transferred by marine organisms, posing a threat to food safety and security.

## 4. Detection and Quantification of Microplastics

Microplastics are usually identified firstly by visual inspection, and based on the particle size, the naked eye, binocular microscopes or scanning electron microscopy are used. Subsequent identification of the polymer type is carried out using Fourier transform infrared (FTIR) spectroscopy and Raman spectroscopy, as visual inspections are not scientific methods and could lead to false negative results [50]. There is considerable challenge in isolating and identifying microplastics depending on the sample type, high pigment content and changes due to extreme weather conditions over time as they complicate spectroscopic analysis, in addition to the requirement of the access to and use of sophisticated machines such as micro-FTIR and micro-Raman [8].

Microplastics are most commonly quantified as the number of items per kg, the number of items per m^3^ and items per individual, and these quantifications are for samples of sediment water and biota, respectively [51]. The sample extracts are counted after visual inspection by a stereomicroscope equipped with a color digital camera, and the number of particles in the form of sludge is given as particles per kg [22]. Pressurized Fluid Extraction (PFE) is a method which can quantify microplastics from various samples of the environment [52].

It can therefore be seen that false positive results can be obtained when identifying microplastics by the naked eye. While the use of equipment is desirable, this may not be always available and it is expensive in most instances, and this is in addition to the requirement of expertise. Therefore, this requires the development of standard, cheap and easy methods, which would require less highly skilled individuals, to be adopted worldwide, so that the accurate detection and quantification of microplastics can be performed in a reproducible manner.

## 5. Sampling and Separation Methods of Microplastics

Three main sampling techniques are generally employed in marine environments and these include selective, bulk and volume reduced. Selective sampling mainly involves taking samples recognizable as plastics by the eyes from the surface of sediments. Bulk sampling collects all the volume of the sample, and plastic samples covered by sediments are collected in this method. In volume reduced sampling, the bulk sample of water or sediment collected is reduced by sieving and filtering, leaving only the sample that is needed [53].

Samples from the water’s surface are generally collected with a trawl net, and samples of the sediment are collected with a grab sampler. Microplastics are extracted from the sample using various techniques, such as density separation, chemical digestion, sieving and filtration. Visual sorting is conducted to identify microplastics based on their morphological characteristics such as size, shape and color [51]. Atmospheric microplastics are usually collected using a special pump [54], and portable active samplers with size selective inlets are used to concentrate airborne particles on fiber filters, and sampling is performed at a flow rate of liter per minute over a specific time duration [55].

Sediment samples collected from the sea are sieved with a 5 mm mesh screen to separate them from macroplastics and other biogenic and anthropogenic particles, and the subsequent treatment involves their homogenization and division into smaller portions. The samples undergo further treatment by drying them in a ventilated oven and further sieving to separate 2 mm and 5 mm microplastics. An optical stereo microscope is used for the counting of larger retained particles that are to undergo identification by attenuated total reflectance (ATR) FTIR spectroscopy [56]. There are shortcomings related to these sampling and separation methods. For instance, data obtained from selective sampling may not always be a true representation of the actual microplastics of the environment studied, visual identification may give false results and trawl size may allow much smaller microplastics to escape. Therefore, this requires a more scientific approach and standardization.

## 6. Characterization Methods of Microplastics

Complex changes, contamination and variations related to size, shape and chemical makeup have made it difficult for the standardized experimental analysis of microplastics [57]. Before tissues are subjected to analysis for microplastics identification, the tissues are first digested to obtain the microplastics. Most studies use nitric acid to digest tissues for microplastic analysis; however, this chemical degrades polyamide in the process [58]. Six different approaches, with varying durations of hours to weeks, using potassium hydroxide, pepsin in hydrochloric acid, nitric oxide, nitric oxide in perchloric acid, sodium hydroxide and peroxydisulfate in sodium hydroxide found that five of the approaches degraded the plastics/poorly degraded the tissue for analysis. The approach using potassium hydroxide at 60 °C for 24 h was found to be more efficient in tissue degradation without much degradation of the plastics polymers being tested, with the exception of cellulose acetate [58]. A newly developed approach with a fast digestion process utilizes a combination of sodium hydroxide for tissue digestion over an hour and sodium iodide for separation, and it was found to recover over 95% of microplastics. However, this is not without changes in size, shape and color [59].

Microplastics are synthesized from a variety of molecules corresponding to their variety, and they are made up of several suites of polymer types. The most commonly synthesized and utilized ones are polypropylene (PP), low density polyethylene (LDPE), high density polyethylene (HDPE), polyvinyl chloride (PVC), polyurethane, polyethylene terephthalate (PET) and polystyrene (PS), which have many varieties and are from many different sources, and they have different sizes, shapes, colors and material type [60]. Thermal extraction/desorption-gas chromatography-mass spectrometry (TED-GC-MS) is a newly developed approach, in which solid water samples are heated to a higher temperature under atmospheric nitrogen which generates decomposed gases that are analyzed with gas chromatography–mass spectrometry (GC-MS) to obtain a chromatogram including mass spectra. The chromatograms enable the identification of the most common microplastics: polyethylene (PE), polypropylene (PP), polystyrene (PS), polyethylene terephthalate (PET), polyamide (PA), polymethylmethacrylate (PMMA) and styrene-butadiene-rubber (SBR) as tire component [61]. Plastic fragments and polymer extracts can also be identified using proton nuclear magnetic resonance (1H-NMR) and attenuated total reflectance Fourier transformed Infrared (ATR-FTIR) [56].

Molecular spectroscopic techniques are used to identify and characterize microplastics: micro-FTIR (for microplastics up to the size of 5–10 µm) or micro-Raman (for microplastics up to the size of 0.2–0.5 µm). The analysis of microplastics is time consuming, making the monitoring process of large quantities difficult. The semi-automated Raman micro-spectroscopy method coupled to static image analysis was validated and found to be faster in the counting and morphological characterization of the microplastics. It is faster because it is easy and quick in localizing, counting and morphological characterization in terms of the size, area, perimeter and shape of the microplastics [62].

A liquid nitrogen cooled mercury cadmium telluride (MCT) detector can be used to increase the speed, resolution and analytical power of micro-FTIR to analyze microplastics as small as 10 microns [63]. Nile red staining (NR staining) followed by fluorescence microscopy and FTIR enhanced the identification of smaller size microplastics, reduced missing of microplastics and time required to identify plastic like particles on spectroscopy [64]. Focal plane array (FPA) based reflectance micro-Fourier-transform (FTIR) imaging is a new method which reduces bias created by the visual inspection of microplastics that has been carried out before analysis, and it has been shown to identify different microplastic types including polyethylene, polypropylene, nylon-6, polyvinyl chloride, and polystyrene [65].

From these discussions, it can be seen that the extraction and analysis of microplastics is a tedious and time consuming process, not only because the tissue digestion and extraction methods are not standardized and can give false information because of the chemical alteration and degradation by the process itself, but also due to the fact that, even after obtaining the microplastics in tissues and other samples such as water and sediment, the spectroscopic confirmatory tests require expensive machines requiring high skill persons. Therefore, this reiterates the need for developing simple, inexpensive, and easy methods that will not necessarily require many skills, and if possible, that can quantify microplastics in food for quality control and food safety and in the environment within a shorter duration, as this may be a necessity in the future looking at the interaction between man and microplastics vis-à-vis the unknown effect between them.

## 7. Bioavailability and Uptake of Microplastics by Aquatic Organisms in Natural Environments

Microplastics are very tiny particles present in our personal care, home, toiletries, gaming, and industrial products such as toothpaste, synthetic clothing, tennis balls, laundry and dish washer pods/tablets, cigarette buts, glitters, wet wipes, tea bags, takeaway cups, cosmetics, hand cleansers, paints, and air blasting, etc. Their durability and non-biodegradable nature together with indiscriminate disposal has made them present in virtually all aquatic environments, and this has made them easily accessible to a wide range of aquatic organisms and their subsequent transfer along the food web [66]. The small particulate size of microplastics is also an important factor that makes them readily available to a large number of aquatic species [67].

The wide distribution of microplastics in aquatic environments makes them readily available to marine organisms even on the deep-sea floor. *Galeus melastomus* at the Bealeric islands were found to have ingested microplastics at a mean value of 0.34 ± 0.07/individual, with most of the ingested plastic being filaments [68]. Wild fish larvae obtained from water samples in the western English channel were found to have ingested microplastics in 22.9% of the studied population, with more than half of the plastics ingested being blue fibers, which are same fibers identified in the water sample [69].

In the Paraiba and Mamanguape estuaries, 205 microplastics in 9% of all fish species sampled were found, with a range of 1 to 4 microplastics ingested by each fish. Fish size was found to not have a correlation with microplastic abundance [70]. A similar study noticed that 36% of the fish sampled contained microplastics [71], and another study showed that 19.8% of fish from the Portuguese coast have plastic waste in their gut [72]. Plastics are said to have a life span of hundreds to thousands of years, and this lifespan is expected to be even higher in deep oceans and other non-surface environments, conferring them the ability to be taken and transferred across the food web. The threats of these plastics include ingestion, choking and the starvation of marine organisms. Plastics also serve as vehicles for the distribution of harmful organisms to a non-native environment, and they carries toxic chemicals and later disintegrate into microplastics which are finally ingested by marine organisms [73].

This indicates that although microplastics are artificially synthesized, they are on the verge of taking over our aquatic ecosystems and have been in aquatic animals across the whole length of the food chain. This requires urgent measures at the level of production and pollution control to save the aquatic ecosystem from disruption.

## 8. Toxic Effect of Microplastics on Aquatic Organisms and Mammals

Microplastics have a varied effect on aquatic organisms and mammals. In aquatic environments, their effect spans across the food chain, negatively affecting growth and reproduction and reducing survival rate. They reduce the consumption of natural prey by larger animals, and as predators they are susceptible to the negative impact of the microplastics [74]. Marine organisms were found to select prey smaller than microplastics [75]; however, some fed on the microparticles more than they did on natural prey [76], whereas some confused them with prey or ingested them during filtration [77]. Studies on mussels from the Norwegian coast showed an average of 1.5 microplastics per individual and correspondingly 0.97 MPs per gram [78].

Microplastics’ effects on marine organisms were earlier focused on entanglement and ingestion, causing various degrees of injury and often mortality. Recently, these microplastics were found to serve as vehicles absorbing contaminants, metals and pathogens from the environment into the organisms, and their interaction produces a synergistic effect to produce a more toxic effect on the organisms [79]. Polystyrene microplastics were found to modulate about 78 proteins in the gills of zebra mussels (*Dreissena polymorpha*), most of which are concerned either directly or indirectly with response to oxidative stress [80].

Although there are limited studies on the effects of microplastics on mammals, they were found to accumulate in animals across the higher trophic level mammalian inclusive. Microplastics was found to accumulate in the liver of mice fed with drinking water and fish containing microplastics. Additionally, neurobehavioral changes including slow locomotion and higher anxiety were observed [81]. Polystyrene microplastics exposure in Wister rats was found to have cardiac toxicity and the levels of troponin I and creatine kinase-MB were elevated, leading to myocardial damage and death, and collagen proliferation in the heart by the induction of the Wnt/β-catenin signaling pathway was observed [82].

Table 2 highlights the effects of microplastics on aquatic organisms and mammals. Most of the studies were conducted on lower aquatic species and the few studies on mammals were mostly on mice and rats as models, the effect of which may not easily be extrapolated to humans. Most of the targeted responses studied focused on oxidative stress, feeding, inflammation, reproduction and mortality rate, without giving much focus on cellular and molecular mechanisms as to how the microplastics affect the organisms.

## 9. Microplastics Exposure Effect on Fish Species

Microplastics may have an effect on various organs of the fish including the brain, liver, gut and gills with a wide range of adverse effects such as vascular injuries, oxidative stress, tumor formation and iono-regulatory disturbances [90]. Intestinal damage and oxidative stress were said to be the main effects of microplastics; where the breakage of enterocytes and villi were demonstrated on Zebrafish, microplastics’ size dependent effect was seen and found to be more with the larger sizes [91]. Inflammation and the accumulation of fat induced in the liver of zebrafish following exposure to polystyrene microplastics of different sizes has been demonstrated, more so oxidative stress was noticed as biomarkers, superoxide dismutase (SOD) and catalase (CAT) were found to be significantly raised. The liver metabolic profile and lipid energy metabolism were also deranged [92]. Similar studies involving gold fish showed evidence of the exfoliation and inflammation of the gut epithelium as well as sinusoidal dilation in the liver [93].

Polystyrene microplastics inhibit the hatchability, decrease hatching time and also suppress the growth of the larva of *Oryzias malastigma* fish [94]. Polyethylene microplastics inhibit oogenesis in zebrafish through up regulation of genes in the intestines and liver that have relation with the aryl hydrocarbon receptor pathway, and abnormal behavior including tail bent downward and seizures was also noticed [95]. Microplastics combine with chemical contaminants and exert a synergistic effect. In addition, they significantly alter homeostasis in the liver, brain, muscles and intestinal tissues [96]. Chronic exposure to polystyrene microplastics in maturing Japanese medaka showed inflamed enterocytes; decreased egg production; histological alteration in the gut, pharynx and spleen; dose dependent glomerulopathy and nephrogenesis [97], and neurotoxicity by the inhibition of acetylcholinesterase activity in the brain of freshwater red tilapia fish following exposure to polystyrene has been demonstrated [98].

Adult zebrafish exposed to polystyrene microplastics for a period of two weeks were found to have increased gut mucus secretion, and mRNA concentrations of IL1a, IL1b, IFNa and their associated proteins, thus indicating the development of inflammation. The composition and richness of gut microbiota in microplastics-exposed zebrafish were changed significantly [99]. Zebrafish exposed to microplastics showed significant alterations in metabolomic and metagenomic profiles in addition to evidence of tissue inflammation and oxidative stress in the gut tissue [100].

Polyethylene and polystyrene microplastics have been found to influence gene expression related to immunity, they also down regulate genes concerned with lipid metabolism and epithelial integrity. This could possibly predispose the fish to pathogenic organisms and also alter their energy metabolism [101].

**Figure 3:**
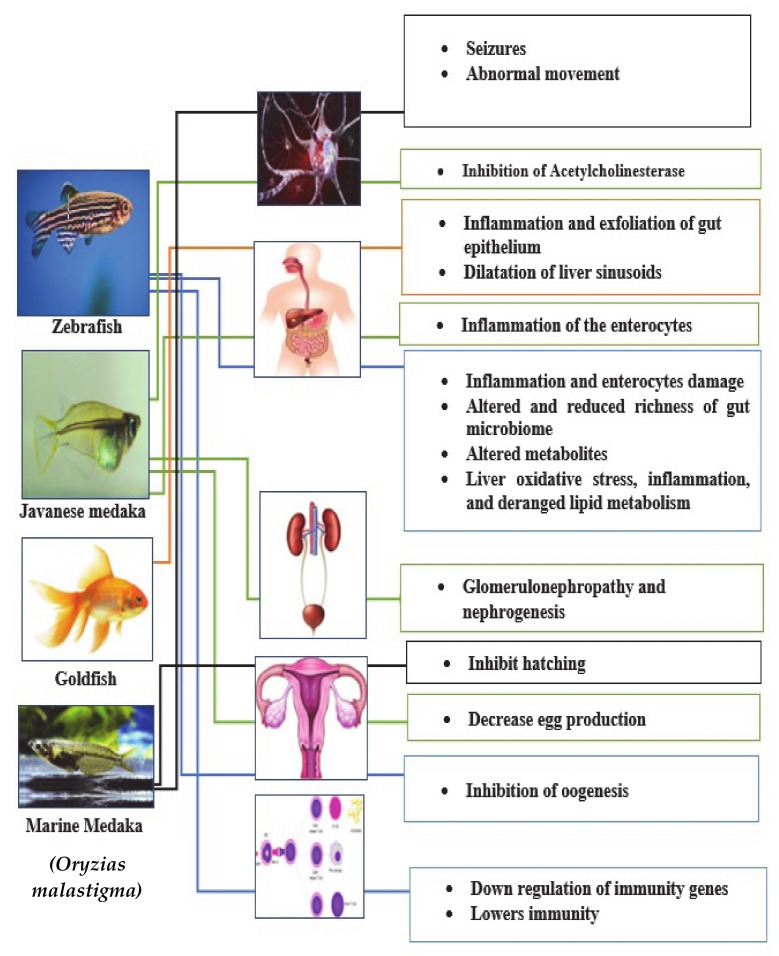
Effect of microplastics on various organ systems of fish species.

As shown in Figure 3 and Table 2, a summary of the varied effects of microplastics on fish species affecting various organ systems demonstrates that most of the studies are mainly limited to zebrafish, with few on *Oryzias* species and goldfish. The studies mainly looked at microplastics’ effect on inflammation and oxidative stress, and only limited studies focused on the gut microbiome and metabolome axis. This clearly reveals the need for extensive studies on different animal models, with the targeted goal of assessing the human health risk of microplastic exposure, and to be able to come up with molecular and metabolic signatures that will possibly aid in the early detection of exposure and health impact on humans.

## 10. Human Exposure Pathways and Health Impact

**Figure 4:**
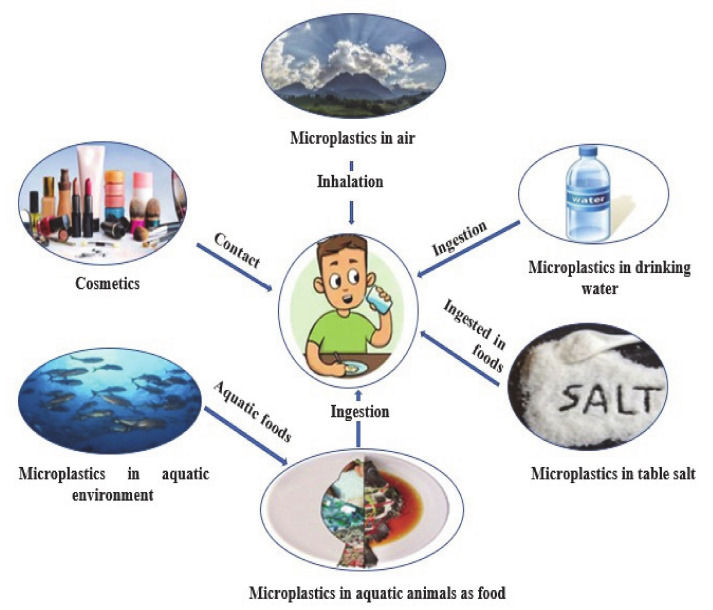
Pathways of human exposure to microplastics.

Seafood consumption has drastically increased over the years. As of 2015, global seafood consumption represents 6.7% and 17% of all protein consumed and total animal protein consumed, respectively. This is representing a major pathway of human exposure to microplastics [102]. Humans are no doubt exposed to microplastics because of the increasing consumption of seafood [103], and also because microplastic pollution has been found almost everywhere. For instance, in every part of East China Sea, microplastics, predominantly the polyester polymer of the fiber type, are found. This is likely at a higher concentration in the higher tropic fish level, mainly in the gills and gastrointestinal tracts [104]. Besides seafood, other sources of human exposure to microplastics have been unveiled, and these include atmospheric air, salt, drinking water and cosmetics, as described in Figure 4. This indicates the need to take appropriate measures to determine possible health risks and mitigating measures.

The gastrointestinal tract of wild caught fish and mussels has been seen to contain microplastics [105]. The contamination of water bodies globally has made microplastics bioavailable in several marine organisms, including those intended for human consumption [106]. In Malaysia, commercial fish intended for human consumption in a seafood market were found to contain microplastics in their internal organs [107], and abiotic sea products such as salts in commercial supermarkets have been reported to be contaminated with microplastics [3]. Microplastics synthesized from polyethylene (PE), polypropylene (PP), polystyrene (PS), polyethylene terephthalate (PET) and polyesters were fed upon by a variety of marine organisms including zooplanktons, mussels, oysters, shrimp and fish, which made them readily available in human food chains through various pathways [9].

The retention capacity of microplastics by the body is largely related to their physiochemical characteristics and the chemical additives used during the manufacturing process [102], and the overall adverse effect resulting from toxic exposure depends on several factors, notably individual susceptibility, hazard control measures, toxic chemical nature and the exposure type [103]. The small size of microplastics and their poor biodegradable property confers them the ability to be ingested by marine organisms and make their way into the food chain, becoming a danger to environmental health, ecological safety and human health [108,109]. Microplastics were said to be ingested through food at an estimate of 39,000 to 52,000 particles per person per year [110]. Another route of exposure to microplastics is through the airways by inhalation, and 26 to 130 airborne microplastics per day are said to be inhaled by an individual [111]. A male with light activity is expected to inhale 272 microplastics per day based on air samples taken using a mannequin [110].

Chemical toxicity may result following the ingestion or inhalation of microplastics by mounting an immune response, but this is not thought to be of much more concern than chronic toxicity that may result from the cumulative effect of exposure over time, and it is speculated that the effect may be dose dependent although there is a lack of sufficient evidence based on the level of exposure. This makes assessing exposure levels a huge gap requiring urgent attention because of the potentiality of microplastics to impact human health [5]. The commercial salts of 128 brands from 38 different countries across 5 different continents were found to contain microplastics, and even though the concentration is much lower compared to those found in aquatic organisms, commercial sea salt is a human exposure source that can produce a potential adverse effect in the long term [112].

Microplastics can translocate into other tissues to induce cytotoxicity and oxidative stress because of their large surface area and their ability to persist, and the difficulty of removing them from tissues enhances their chronic inflammatory effect and the risk of cancer. As particulate matter, they have the potential to increase the risk of neurodegenerative and immune diseases [113].

Microplastics indirectly pose a serious hazard to human health by their alteration of the aquatic microbial community through gene transfer, and they also enhance the spread of microbial resistance [114]. An increase in the utilization of antibiotics in agriculture and related products has led to their presence in aquatic environments and the emergence of antibiotic resistant bacteria (ARB). Microplastics in water environments serve as a vehicle to microbes including ARB, thereby making them likely to be imported to aquaculture and possibly to humans through the food chain after a long period of time [115]. The chronic effect of microplastic accumulation in the cells and tissues of aquatic organisms poses a potential hazard to humans, as the subsequent ingestion of microplastics can lead to chromosomal alteration, resulting in infertility, obesity, and cancer [116].

The hydrophobic and high surface to volume ratio of microplastics make them capable of adsorbing and accumulating persistent organic pollutants [117]. Microplastics were demonstrated to serve as a carrier of decabromodiphenyl ether (BDE-209), and the effect of BDE-209 was increased where it negatively affected phagocytosis and structural damage in some tissues [118]. The acute toxicity of methamphetamine in association with microplastics is significantly increased through oxidative damage and apoptosis in snails, and this may have an impact through the entire aquatic food chain [119]. Nylon microplastics have been found to adsorb three divalent metals (copper, nickel and zinc), and these metals are common pollutants in water environments which now have microplastics as carriers, thus showing the toxicity potential of microplastics together with other compounds [120]. Microplastics also serve as carriers of bisphenols and may provide an important route for increasing health risks. This is because, in a simulated intestinal environment, polyvinyl chloride (PVC) attached with bisphenol easily released the bisphenol manyfold in aquatic environments, making it readily bio accessible [121]. Bisphenol A (BPA) is widely used in industries, despite being recognized as an endocrine disruptor. With studies linking it to diseases such as obesity, infertility, diabetes and cancer, it is used to produce epoxy polymers for coating metal cans, and it is also incorporated in plastics including bottled water as polycarbonate [122]. BPA can cause single strand and double strand DNA breaks, and its metabolic byproduct was found to be a DNA adduct. Additionally, BPA interferes with many signaling pathways such as NFκB, JNK, MAPK, ER and AR, which leads to disease and tumor development [123]. Following regulations on the use and production of BPA due to its adverse effects and roles in disease development, several analogues including BPAF, BPB, BPF, and BPS have been produced as alternatives; however, these analogues were found to cause cytotoxicity, genotoxicity, reproductive toxicity, dioxin-like effects, and neurotoxicity in animal studies, and they were also found to be present in abiotic environment and human urine in certain regions [124]. PVC is easily degradable, releasing bisphenols and PVC microplastics, which in turn take bisphenols and pollute the aquatic ecosystem. BPA and its analogues were seen to have a positive effect on hydrophobic interactions and non-covalent hydrogen and halogen bonds with polyvinyl chloride, and this promotes the adsorption of bisphenols on PVC [125]. BPA was detected in 70% of the landfills investigated at a concentration exceeding the LC_50_ levels of aquatic biota. The source is said to probably be from landfill plastic waste [126]. Humans have various sources of exposure to BPA, including its release from food packages and the repeated used of polycarbonate containers such as baby bottles, air, drinking water, soil and dust [127].

Even though these microplastics are well documented in readily available food and water meant for human consumption in the market, little is known about their possible acute and chronic effects with regards to food safety and human health, especially since microplastics are potential vectors of many toxic compounds such as heavy metals and bisphenols in addition to serving as carriers of ARB. This clearly shows the danger of microplastics alone and in relation to other compounds and organisms, which can affect the environment, aquatic organisms and humans, who can be exposed through several routes. This necessitates further investigation to find ways to address this problem. Table 3 summarizes the presence of microplastics in food and water meant for human consumption.

## 11. Solution and Policy Development to Microplastic Pollution

There is a myriad of challenges in controlling the menace of plastic pollution. In 1967, a call was made to the world communities to address the problems of the seas and oceans, and a convention with a similar mandate by the United Nations Environment Assembly on plastic pollution was also held in 2017. Although these meetings provide the foundation in offering solutions to the problem of plastic pollution by addressing both governments and non-governmental organizations, consensus at an international level is often delayed in the process of being developed, criticized, and often not agreed upon to be of positive outcome. Therefore, this necessitates the need for evidenced based and tailored scientific research and collaboration between the government and other sectors to address the utilization and discharge of plastics [134].

Plastics have been under critical observation in the environment for a long time because their production had surpassed other man made materials, yet there is an apparent lack of extensive global information, especially with respect to their final fate after utilization [135]. In isolated circumstances, the worldwide governance on plastic is improving, and while many companies including Toyota, Walmart and Proctor and Gamble disposed of waste to landfill, there is also considerable global momentum in research, activism, policies and regulations, such as banning plastics for grocery bags and microbeads in consumer products. However, the plastic making its way into the ocean is forecast to be doubled by 2025. This is due to the difficulty in governance posed by the wide distribution, intrinsic durability and movement of microplastics. Even more, increased production, global utilization, the diversification of pollution source and international trade also render governance a tedious task to accomplish. Despite the mounting pressure, authorities are broken, international institutions are not strong, regulations are not uniform and policies and business-oriented solutions are badly organized with regards to plastic pollution. These necessitate the need for the local regulation of industries as well as international treaties that will strengthen the local reforms [136].

Many countries around the world have signed into law taxing plastics bags and in some cases banning their use. In the United States, many states and local governments have tackled plastic pollution, yet there is no national plastic policy in place due to fervent support of the plastic industry. A holistic multidimensional approach that will give hope to consumers to reduce plastic use is needed [137].

Policies and strategies aimed at controlling microplastic pollution should focus on two key areas, which include controlling the source of pollution and remediation, and microplastic pollutant clean up. However, this is obtainable in few countries, and includes policies such as the microbeads Free Water Acts (2005) of the U.S. government, prohibiting the sale of personal care products containing microbeads effective in 2017, promoting the use of polylactatide (PLA) and polyhydroxyalkanoates (PHA), which are biodegradable alternatives to traditional plastic polymers, enhancing the recycling of plastics, improving the use of plastics as an energy source and synthetic crude, strengthening and improving waste water treatment facilities to effectively separate and prevent microplastics getting into rivers and oceans, and researching and developing bioremediation technologies such as the biodegradation of microplastics using microbes [9]. However, the microbeads Free Water Act is faulted for having a narrow scope and for not encouraging biodegradable options that will solve the larger issue of plastic pollution in the environment [138]. Advanced waste water treatment technologies should also be utilized, and this includes: disc filters (DF), rapid sand filters (RSF), dissolved air flotation (DAF) and membrane bioreactors (MBR) as they are found to be effective in removing >95% of microplastics (>20 mm) from effluents [139].

In China, plastic pollution is classified under solid waste and managed by the Law on the Prevention and Control of Environmental Pollution by Solid Wastes (LPCEPSW), which regulates waste disposal sites, prohibits the dumping of plastics in rivers, lakes and reservoirs, and promotes circular economy. In addition to the national law, China has many state laws that regulate plastic waste disposal, but the laws are not very effective and tedious to enforce as dumping is still ongoing in rural areas, plastic bags are still used in markets and microbeads in personal care products are still allowed, despite being banned in countries like the US, Canada, New Zealand, the Netherlands and Ireland [140]. 

Tax has been imposed on single use plastic bags, but this could not change consumer behavior, especially since these regulations in some countries are only particular to some regions—this is in addition to the plastic industries’ resistance against the taxation or ban on plastic use. Agreements at an international level to curb the menace of plastic pollution are poorly coordinated and have resulted in disjointed governance systems. The United Nations Environment Assembly (UNEA) had three sessions in relation to plastic pollution, the recent was held in December 2017 with a focus on adopting new practices, identifying possible obstacles and grey areas, and coming up with possible solutions. It also set up a committee to create response strategies to combat plastic pollution through government policies and voluntary options. However, these measures did not emphasize the causes of pollution and the implementation of the policies that will address the problem right from the increase in production to the impacts of plastic till it reaches its complete life cycle [141].

To combat microplastic pollution, proactive intersectoral involvement is required. A global mindset and local action are paramount to decrease the threat. Laws and education regarding the environmental effects of microplastics are needed to curb the menace of plastic pollution. Researchers and the public domain should shoulder the responsibility of ensuring that governments and businesses imbibe an attitudinal change towards the threat [142]. Products containing microplastics should be labeled and shared to consumers and alternatives to these products which are environmentally friendly and sustainable should be communicated. This has been demonstrated by campaigns aimed at stopping the use of microbeads through emailing consumers by major cosmetic brands with non-plastic alternative products containing walnut husks, oatmeal, and granulated sugar. Mobile applications have also been developed by some companies such as Dutch NGOs, the plastic Soup Foundation and the North Sea Foundation, which give information on microplastic beads-containing products to allow the consumers to make an informed choice [19].

Plastic pollution remains a problem needing solutions, and many approaches are being tried to combat it as a global threat. One of the areas being explored is the use of microorganisms capable of degrading plastics, especially those obtained from Antarctic cold regions; however, the complex interaction between the two is poorly understood [1]. In 2018, the Malaysian government came up with a roadmap for zero single plastic use, a planned and targeted policy that will span through 2030. Malaysia has around 1300 industries that manufactures plastics and ranked 8^th^ out of the top 10 countries with poorly managed plastic waste. The road map, which will be in phases, includes measures such as: setting strong institutions on this aspect; the Communication, Education and Public Awareness (CEPA) Plan; the use of biobags to replace plastic bags; a levy on plastics to manufacturers; a pollution charge on single-use plastic bags; R&D funding for alternative environmentally friendly products; regional cooperation on marine plastic waste, among other measures. However, these measures are being faced with many challenges, such as poor awareness, low recycling rate, the high cost of alternative non plastic products, enforcement hitches at the local level and the need for an integrated waste management approach that can convert the biodegradable alternatives to products such as fertilizers, energy and animal feed by downstream industries, thereby serving as a trigger for waste to wealth intervention [143].

In 2020, Australia introduced the Recycling and Waste Reduction Bill, which is a legislation incorporating an existing product stewardship act from 2011. The law provides a flow chart of waste management and recycling within the country, and the law bans the export of waste material including plastic, paper and glass, as such taking responsibility of its waste [144].

Governance issues with regards to plastic litter are complicated and challenging because they cut across international boundaries. Reversing the environment back to a pre-plastic era is also a challenging task. Thus, this requires an all-encompassing approach involving scientists, community members and strategies in plastic marketing that will reduce the global plastic menace [145]. These policies and regulations are only in a few countries, and even when they are in place they are poorly enforced. In some instances, they do not involve rural regions and they are limited by international boundaries, even though this is a global problem affecting virtually all water bodies. There is also poor consumer education related to the effects of pollution, which ultimately will require scientific evidence to support the consequent negative health effects of the menace to allow consumers to make informed decisions. Table 4 summarizes some of the laws, policies and strategies put in place by some countries and organizations aimed at tackling the menace of plastic pollution. This clearly shows a huge gap in regulations around the globe as only a limited number of countries are proactive, even so with huge challenges in implementation.

## 12. Conclusions 

Microplastics have become part of our marine environment for decades and they have been predicted to increase in an order of magnitude in the years to come. These plastics do not only affect the environment and aquatic organisms but also end up in food meant for human consumption, posing a potential threat to food safety and security. This necessitates the need for extensive studies that will provide the possible health effects of this pollutant and allow us to have an insight into its toxicity mechanisms, and to determine biomarkers that could be used as a sign of toxicity in humans. Standardized methods of detection and quantification methods that are fast, easy, and reliable should also be developed. Policies should also be reinforced and must be at a global scale. Awareness, education, and communication involving governments, industries, non-governmental organizations, and consumers should be initiated to allow for collective action and informed choices to curb microplastic pollution.

## Figures and Tables

**Table 1:** Microplastic pollution of waters and sediments in different regions of the world.

Location	Density	Size	Polymer Type	References
Milwaukee River basin, Wisconsin, USA	1.1 g/cm^3^	0.355–4.749 mm	LDPP, PET	[32]
Hudson River, New York State, USA	0.98 items/L	1.000–4.749 mm	PET, PP	[33]
Rhine riverbed, Koblenz, Germany	0.26–11.07 × 10^3^/kg weight	11−5033 μm	AC, PU, APV, PE, EPDM, PES	[34]
River Rhine, River Main, Germany	228–3763 particles/kg	63–5000 μm	PE, PP, PS, PET, EPDM, PVC	[35]
Saigon River, Vietnam	172,000–419,000 Items/m^3^	50–250 μm	PE, PP, PES, PET	[36]
Snake and lower Columbia rivers	0.014–5.405 items/L	100–333 µm	PP, PE, PET, PES	[37]
Italian coast, Italy	0.641 to 0.119 items/m^3^	≤333 µm	PP, PE, PET, PES, EVA	[38]
Greater Melbourne Area and the Western Port area, Australia.	0.06 to 2.5 items/L	1.26 ± 0.93 mm	PES, PP, PE, PA	[39]
Bohai Sea China	0.33 ± 0.36 m^3^	0.3–5 mm	PE, PP, PS, PET	[40]
Yangtze River Basin China	0.5–3.1 items/L	0.25–1 mm	PES, PP, PE	[41]
Greenland Sea	0.81–4.52 particles m^−3^	0.5–4.5 mm	PES, PE	[42]
Hong Kong Marine waters	413.38 particles m^−3^	0.2–4.9 mm	PP, PE, SAN	[43]
China coastal waters	0.68 to 6.44 particles/L	0.25–1 mm	PET, RY, PE, PVC, PP	[44]
Southern Baltic sea	25 to 53 particles/kg dry weight	0.1–5 mm	EPM, PVC, VCE, PAN, PVA, PES, EVA, PE	[45]
Bohai Sea China	2.0–17.0/50 g dry weight	66.25–4982.59 µm	RY, PE, PET, PP, PA	[46]
Northern Yellow Sea China	4.0–14.0 particles/50 g dry weight	66.25–4982.59 µm	RY, PE, PET, PP, PA	[46]
Southern Yellow Sea China	2.0–7.0 items/50 g dry weight	66.25–4982.5 µm	RY, PE, PET, PP, PA	[46]
Oman sea	138.3–930.3 particles/kg	100–1000 μm	PE, PP, PA, PET, PVA, PS, PVC	[47]
Canterbury’s coastlines	0–45.4 particles/Kg of dry sediment.	0.5–1 mm	PS, PE, PP	[48]
Xiangshan Bay, China	Water: 8.9 ± 4.7 items/m^3^Sediment: 1739 ± 2153 items/kg	1.54 ± 1.53 mm 1.33 ± 1.69 mm	PE, PP, PS	[21]
River Thames, UK	66 particles/100 g	1 mm–4 mm	PP, PES, PAS	[49]

PE: Polyethylene; PP: polypropylene; PS: polystyrene; PET: polyethylene terephthalate; PVC: polyvinylchloride; PES: Polyester; PA: Polyamide; EVA: ethylene vinyl acetate; PA: nylon; EPM: ethylene-propylene rubber; VCE: Poly(vinyl chloride-ethylene); PAN: Polyacrylonitrile; PVA: Polyvinyl Alcohol; PAS: polyarylsulfone; PU: polyurethane: AC: Acrylate; APV: acrylates/polyurethane/varnish cluster; EPDM: ethylene-propylene- diene rubber; LDPP: low-density polyethylene (LDPE).; RY: Rayon; SAN: styrene acrylonitrile.

**Table 2:** Toxic effects of microplastics on organisms.

Type	Size (μm)	Concentration	Organisms	Tissue	Biomarker(s)	Response	References
PS	1 and 10	50 mg/L	Zebra mussels (*Dreissena polymorpha*)	Gills	Proteome	Change in protein involved in oxidative stress, ribosomal function, energy metabolism, cellular trafficking, RNA binding and cytoskeleton	[80]
PE	35.46	500 mg/mL	Mice	CNS	Stress locomotion	Reduced locomotion Anxiety	[81]
PS	0.5	0.5, 5, 50 mg/L	Wistar rats	Heart	Troponin I Creatinine-kinas MB	Increase troponin I and creatinine-kinase MB Myocardial damage and apoptosis by induction of oxidative stress Collagen proliferation in heart by activation of Wnt/β-catenin pathway	[82]
PS	5 and 20	0.1 mg/day	Mice	Liver	ATP, LDH, SOD, AChE	Decrease ATP, LDH and AChE Increased GSH-Px and SOD	[83]
PS	5	100 and 1000 µg/L	Mice	Gut, liver, and feces		Gut damage, metabolic disorders, microbiota dysbiosis	[84]
PE PS	<100	20 gm/L 0.5 mg/L 5 mg/L	*Mytilus galloprovincialis*	Gills, digestive glands, haemolymph	Immune cells functions NNRT, AChE, DNA MN, NA	Immunotoxicity, neurotoxicity, genotoxicity, changes in gene expression profile	[85]
PE	<400	0.02 gm/L 0.08 gm/mL 0.04 gm/mL 0.08 gm/mL	*Hydra attenuata*		Feeding habit	Reduced feeding	[67]
PS	10	1 × 10^5^ particles/L	Medaka (*Oryzias melastigma*)		Mortality, growth and fecundity	Significant mortality, reduction in growth and egg production	[86]
PS	0.5	40,000 μg/L	*Eriocheir sinensis*	Liver		Inhibits growth Damage and oxidative stress induction in hepatopancreas	[87]
PET	62–1400		Fresh water crustacean (*Daphnia magna*)	Gut	Mortality	Increased mortality Accumulation of PET in the gut	[88]
PS	58	0.25–2%	Earthworms (*E. foetida)*		Growth and mortality	Significant inhibition of growth and mortality	[89]

SOD: Superoxide dismutase; CAT: catalase; GSH-Px: glutathione peroxidase; GST: glutathione S-transferase; GR: glutathione reductase; GSH: glutathione; NNRT: non-nucleoside reverse transcriptase; AChE: acetylcholinesterase; DNAB: DNA strand breaks; MN: micronuclei frequency; NA: nuclear alterations; PE: Polyethylene; PS: polystyrene; PET: polyethylene terephthalate; CNS: central nervous system; ATP: adenosine triphosphate; LDH: lactate dehydrogenase; MB: myocardial band.

**Table 3:** Microplastics in commercial food products meant for human consumption.

Products/Country	Concentration	Plastic Polymer	References
Bivalves/China	2.1 to 10.5 items/g 4.3 to 57.2 items/individual	fibers, fragments, and pellets,	[128]
Commercial fish/Malaysia	56 particles/11 fish	PP, PE, PET	[107]
Commercial salt/China	550−681 particles/kg in sea salts, 43−364 particles/kg in lake salts, and 7−204 particles/kg in rock/well salts	PET, PES, PE, PB, CP, PP	[3]
Commercial salt from Australia, France, Iran, Japan, Malaysia, New Zealand, Portugal and South Africa	1 to 10 microplastics/kg	PET, PE, PP, PET	[129]
Commercial mussels/UK	1.4 items/g	PP	[106]
Dried commercial fish/Malaysia	0–3 particles/fish	PP, PE, PET, PS, PA	[130]
Commercial fish/Mondego estuary	1.67 ± 0.27 (SD)	PP, PAN, PE, polyamide 6-nylon	[131]
Commercial molluscs from the lagoon of Bizerte (Northern Tunisia)	703.95 ± 109.80 to 1482.82 ± 19.20 items/kg	PE, PP	[132]
Returnable water Single plastic bottled water Beverages All in grocery stores Germany.	118 ± 88 particles/L 14 ± 14 particles/L 11 ± 8 particles/L	PET, PP	[133]

PE: Polyethylene; PP: polypropylene; PS: polystyrene; PET: polyethylene terephthalate; PES: Polyester; PA: Polyamide; PA: nylon; PAN: Polyacrylonitrile; PB: Poly 1-butene; CP: cellophane.

**Table 4:** Laws, policies, and strategies aimed at curbing plastic pollution.

Country/Agency/Company	Policy/Strategy	Functions	References
Australia	Recycling and Waste Reduction Bill (2020)	Banning of plastic export. Provides flow chart of waste management and recycling.	[144]
China	Law on the Prevention and Control of Environmental Pollution by Solid Wastes (LPCEPSW)	Regulates waste dumping sites. Prohibition of plastic dumping in rivers, lakes, and reservoirs. Promotes circular energy	[140]
Malaysia	Road map for zero single plastic use	Taxation on single plastic use bags and plastic manufacturers. Communication, education and public awareness. Research and development on alternatives such as biobags.	[143]
USA	Microbeads Free Water Acts (2005)	Prohibition of sales of personal care products containing microbeads	[9]
United Nations Environment Assembly (UNEA)	To identify obstacles, grey areas and to adopt new strategies	Setting a committee to create response strategies. Strategies should target government policies and voluntary options to combat plastic pollution.	[141]
Plastic soup foundation and North Sea foundation	Information on microplastic containing products	Allows consumers to make an informed choice	[19]
Toyota, Walmart, Proctor and Gamble	Efficient plastic waste disposal	Disposing plastic waste to land fill	[136]
